# Supplementary material for: Secoiridoid Glucosides and Anti-Inflammatory Constituents from the Stem Bark of Fraxinus chinensis
Source: Molecules. 2020 Dec 14;25(24):5911. doi: 10.3390/molecules25245911 (PMC7764309; doi:10.3390/molecules25245911)
Supplement: Supplementary file 1 [file molecules-25-05911-s001.pdf]

Supplementary data

Secoiridoid Glucosides and Anti-inflammatory Constituents from the Stem Bark of *Fraxinus chinensis*

Hao-Chiun Chang [1,2,†], Shih-Wei Wang [3,4,†], Chin-Yen Chen [5], Tsong-Long Hwang [6,7,8], Ming-Jen Cheng [9], Ping-Jyun Sung [4,10], Kuang-Wen Liao [2,11] and Jih-Jung Chen [12,13,*]

1 Department of Orthopaedics, Mackey Memorial Hospital, Taipei 10449, Taiwan; Changhaochiun@gmail.com
2 Ph.D. Degree Program of Biomedical Science and Engineering, National Chiao Tung University, Hsinchu City 30068, Taiwan
3 Department of Medicine, Mackay Medical College, New Taipei City 25242, Taiwan; shihwei@mmc.edu.tw
4 Graduate Institute of Natural Products, Kaohsiung Medical University, Kaohsiung 80708, Taiwan
5 Graduate Institute of Pharmaceutical Technology, Tajen University, Pingtung 90741, Taiwan; jjc8506674@gmail.com
6 Graduate Institute of Natural Products, School of Traditional Chinese Medicine, College of Medicine, Chang Gung University, Taoyuan 33303, Taiwan; htl@mail.cgu.edu.tw
7 Research Center for Chinese Herbal Medicine, Research Center for Food and Cosmetic Safety, Graduate Institute of Health Industry Technology, College of Human Ecology, Chang Gung University of Science and Technology, Taoyuan 33303, Taiwan
8 Department of Anesthesiology, Chang Gung Memorial Hospital, Taoyuan 333, Taiwan
9 Bioresource Collection and Research Center (BCRC), Food Industry Research and Development Institute (FIRDI), Hsinchu 30062, Taiwan; cmj@firdi.org.tw
10 National Museum of Marine Biology and Aquarium, Pingtung 94450, Taiwan; pjsung@nmmba.gov.tw
11 Institute of Molecular Medicine and Bioengineering, National Chiao Tung University, Hsinchu City 30068, Taiwan; liaonms@g2.nctu.edu.tw
12 Faculty of Pharmacy, School of Pharmaceutical Sciences, National Yang-Ming University, Taipei 11221, Taiwan
13 Department of Medical Research, China Medical University Hospital, China Medical University, Taichung 40402, Taiwan
* Correspondence: chenjj@ym.edu.tw (J.-J.C.); Tel.: +886-2-2826-7195
† Authors have contributed equally in this manuscript.

Contents

Figure S1. ESI-MS spectrum of **1**.. S4

Figure S2. HR-ESI-MS spectrum of **1**.. S4

Figure S3. ^{1}H NMR spectrum (CD_3OD, 500 MHz) of **1**... S5

Figure S4. ^{13}C NMR spectrum (CD_3OD, 125 MHz) of **1**.. S5

Figure S5. DEPT spectrum of **1**.. S6

Figure S6. ^{1}H-^{1}H COSY spectrum of **1**..S6

Figure S7. NOESY spectrum of **1**..S7

Figure S8. HMBC spectrum of **1**... S7

Figure S9. HSQC spectrum of **1**.. S8

Figure S10. ESI-MS spectrum of **2**...S8

Figure S11. HR-ESI-MS spectrum of **2**... S9

Figure S12. ^{1}H NMR spectrum (CD_3OD, 500 MHz) of **2**... S9

Figure S13. ^{13}C NMR spectrum (CD_3OD, 125 MHz) of **2**...S10

Figure S14. DEPT spectrum of **2**...S10

Figure S15. ^{1}H-^{1}H COSY spectrum of **2**...S11

Figure S16. NOESY spectrum of **2**...S11

Figure S17. HMBC spectrum of **2**.. S12

Figure S18. HSQC spectrum of **2**... S12

Figure S19. ESI-MS spectrum of **3**...S13

Figure S20. HR-ESI-MS spectrum of **3**..S13

Figure S21. ^{1}H NMR spectrum (CD_3OD, 500 MHz) of **3**... S14

Figure S22. ^{13}C-NMR spectrum (CD_3OD, 125 MHz) of **3**..S14

Figure S23. DEPT spectrum of **3**...S15

Figure S24. ^{1}H-^{1}H COSY spectrum of **3**...S15

Figure S25. NOESY spectrum of **3**... S16

Figure S26. HMBC spectrum of **3**... S16

Figure S27. HSQC spectrum of **3**... S17

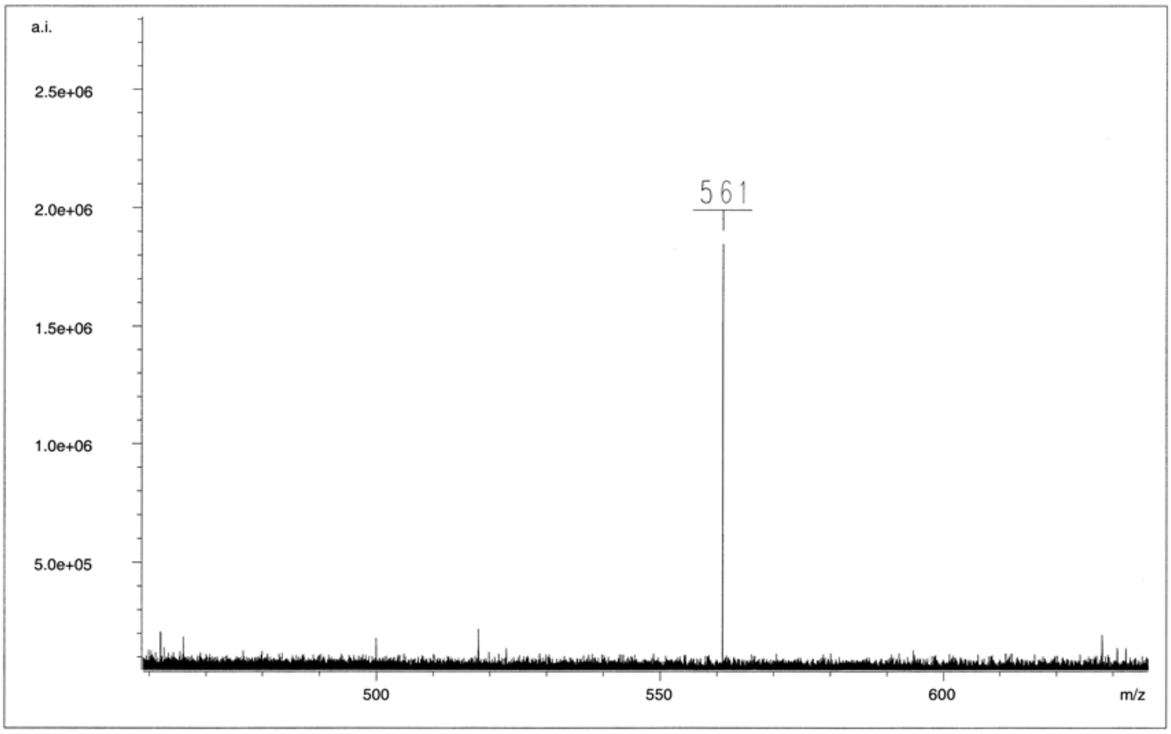

Figure S1. ESI-MS spectrum of 1.

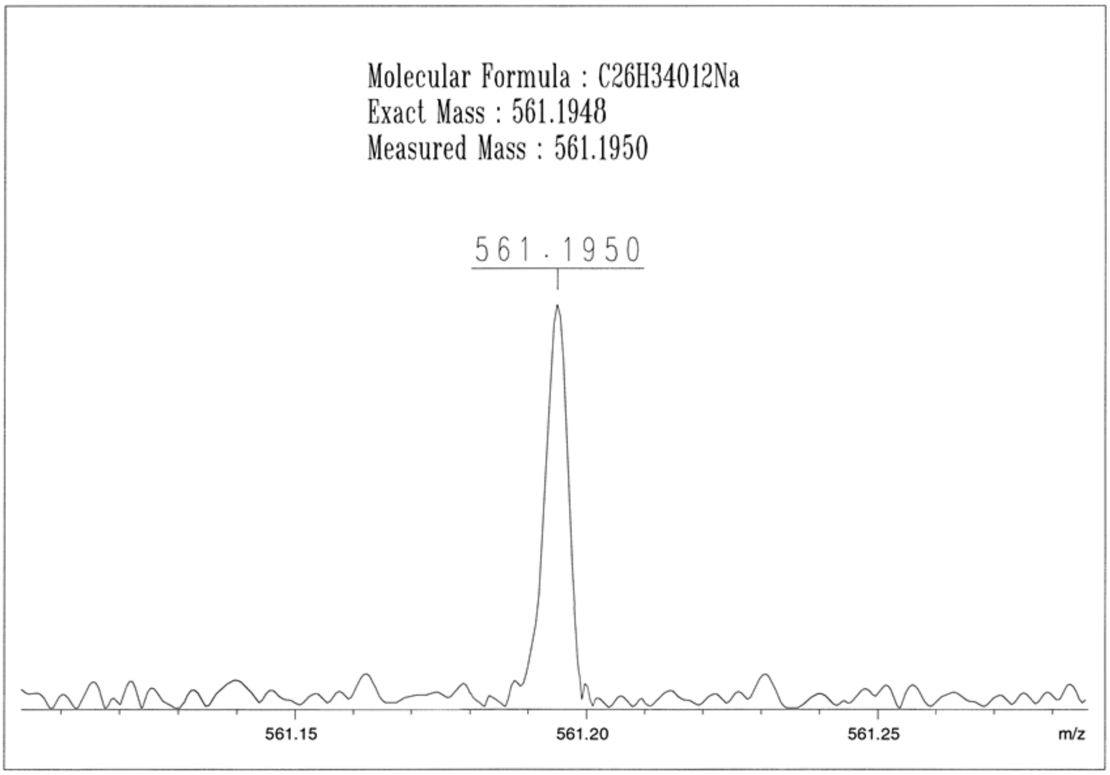

Figure S2. HR-ESI-MS spectrum of 1.

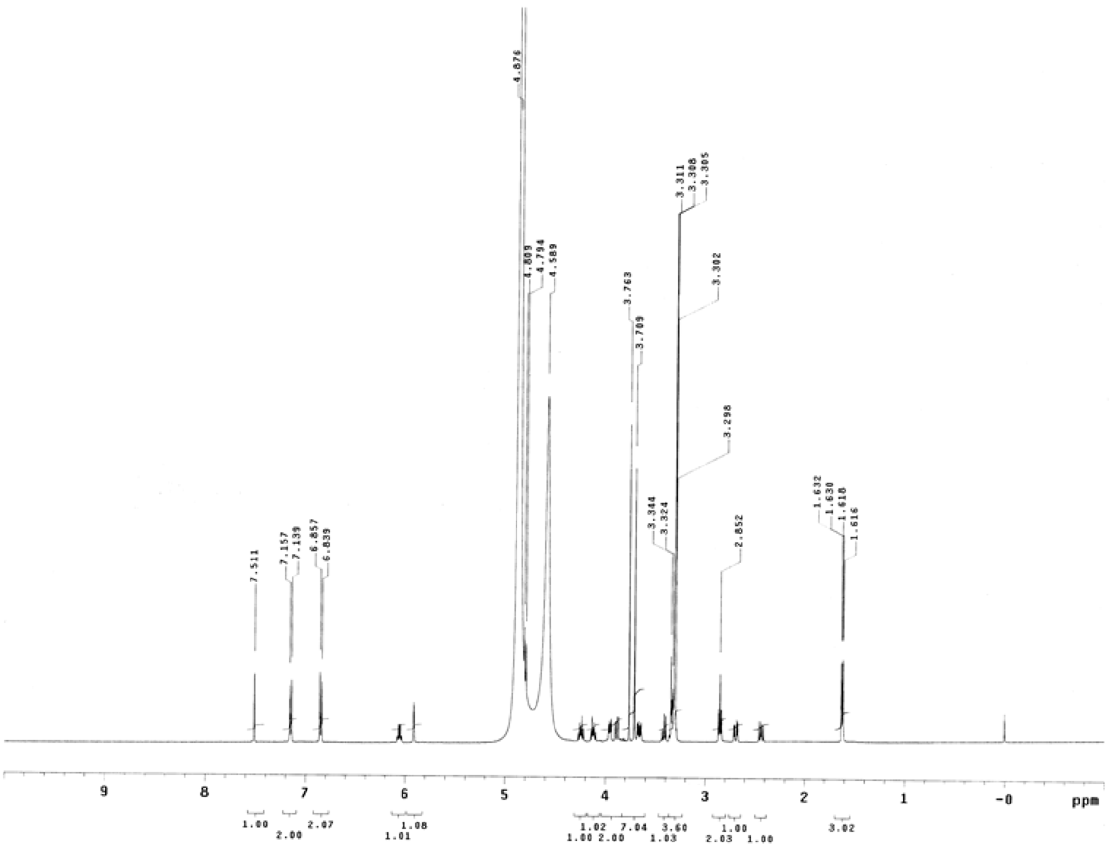

Figure S3. ^{1}**H-NMR spectrum (CD$_3$OD, 500 MHz) of 1.**

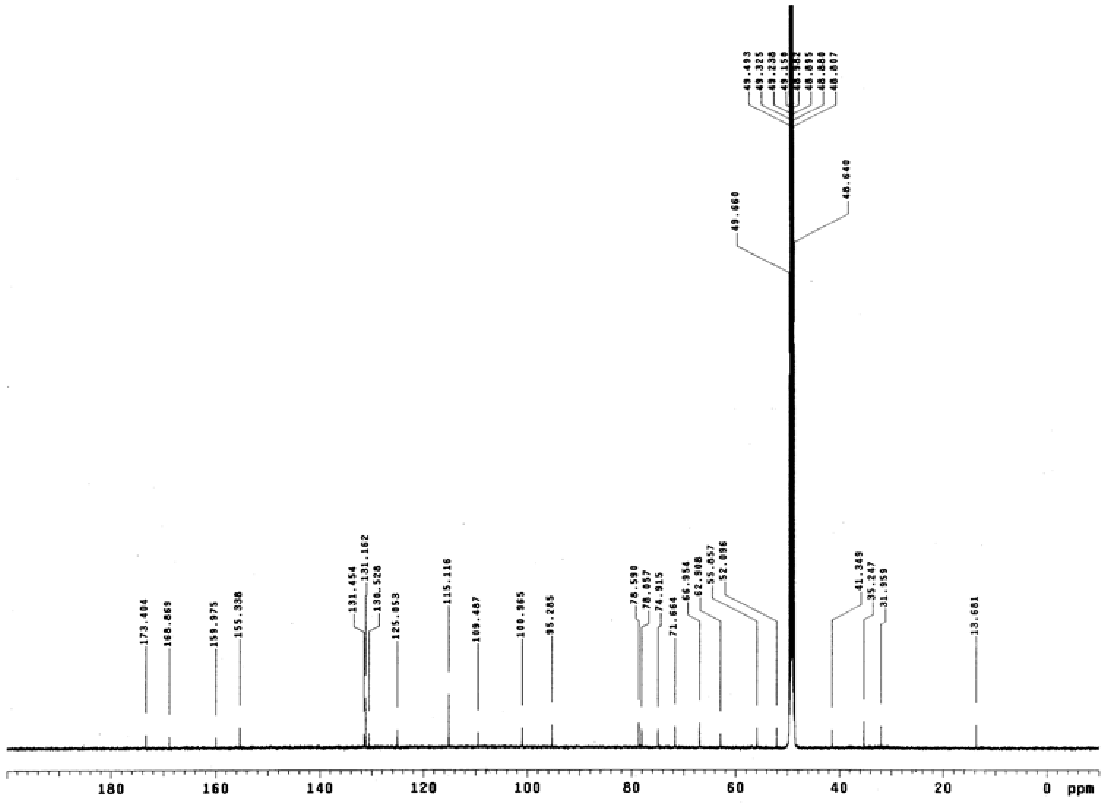

Figure S4. ^{13}**C-NMR (CD$_3$OD, 125 MHz) spectrum of 1.**

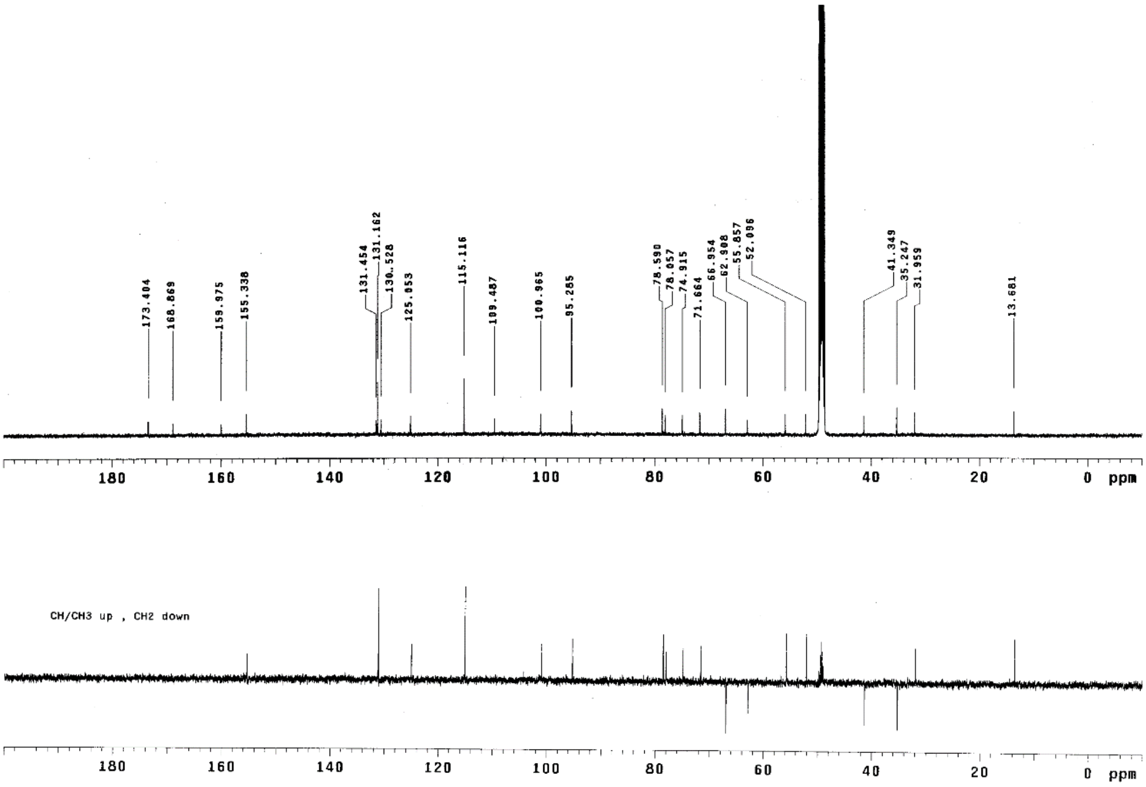

Figure S5. DEPT spectrum of 1.

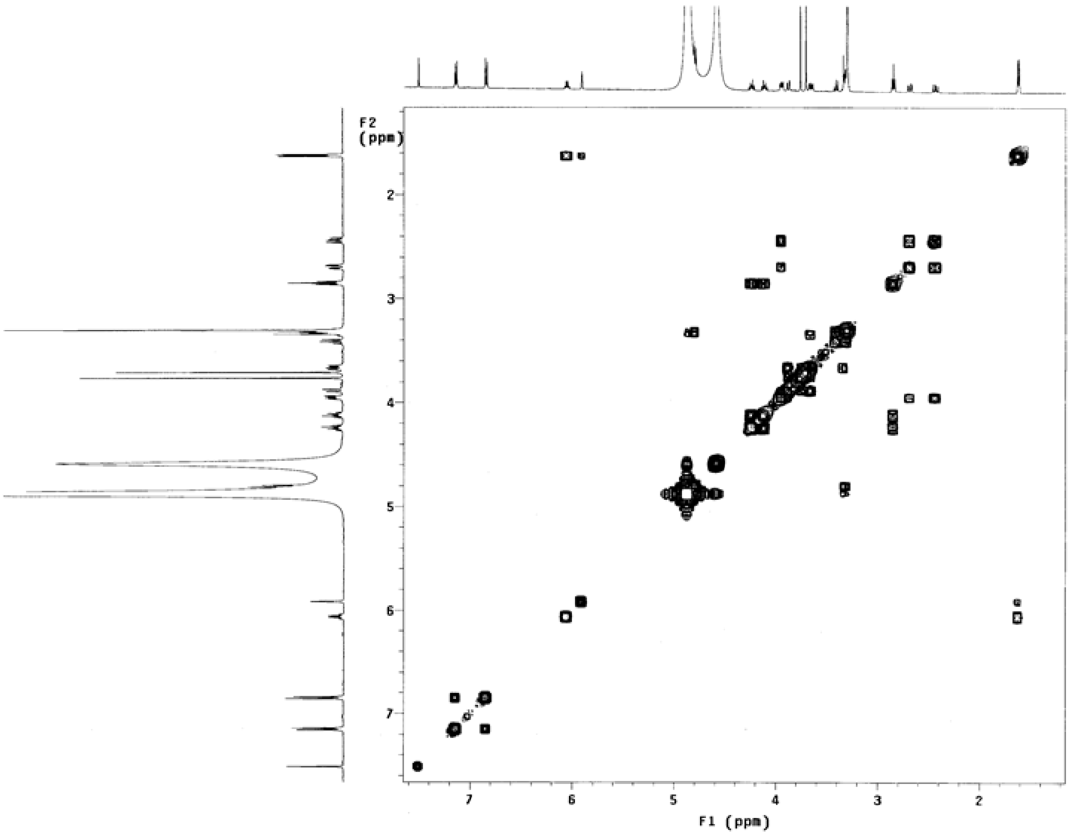

Figure S6. ^{1}H–^{1}H COSY spectrum of 1.

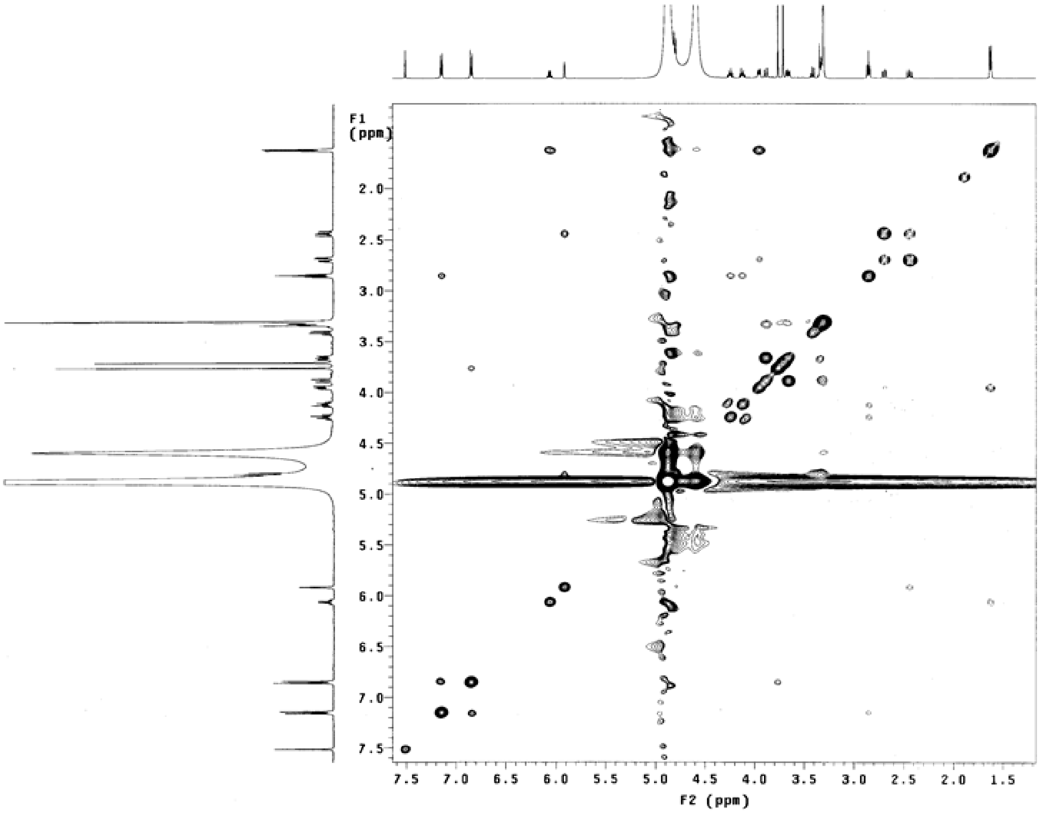

Figure S7. NOESY spectrum of 1.

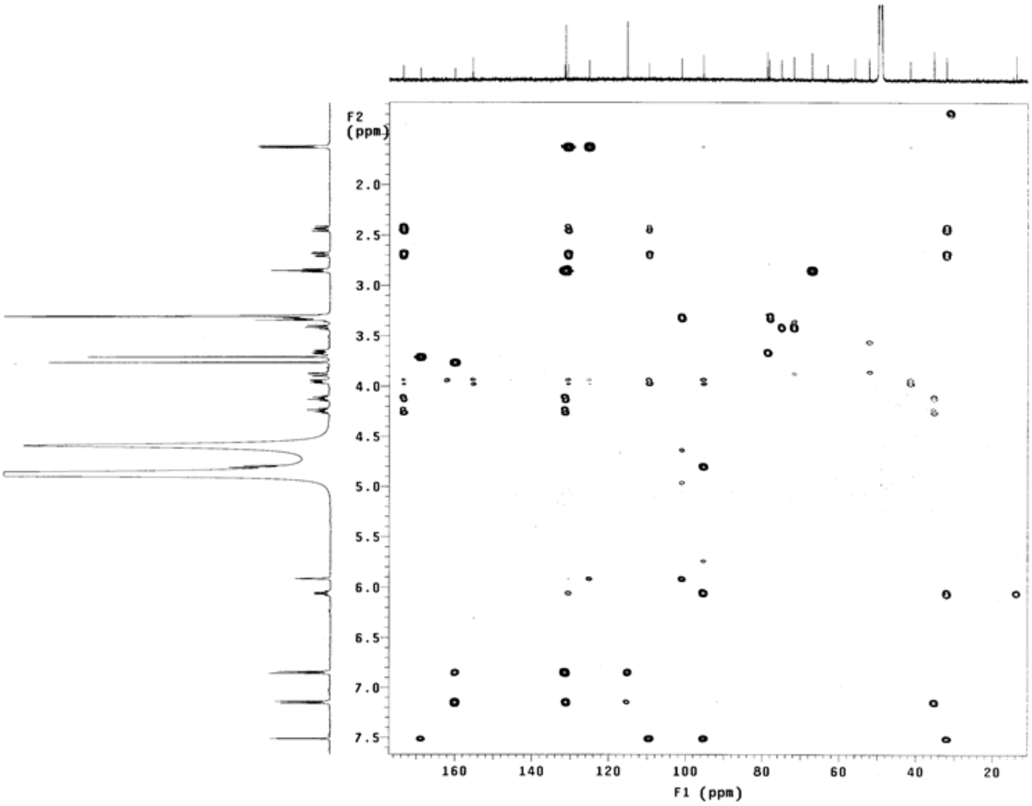

Figure S8. HMBC spectrum of 1.

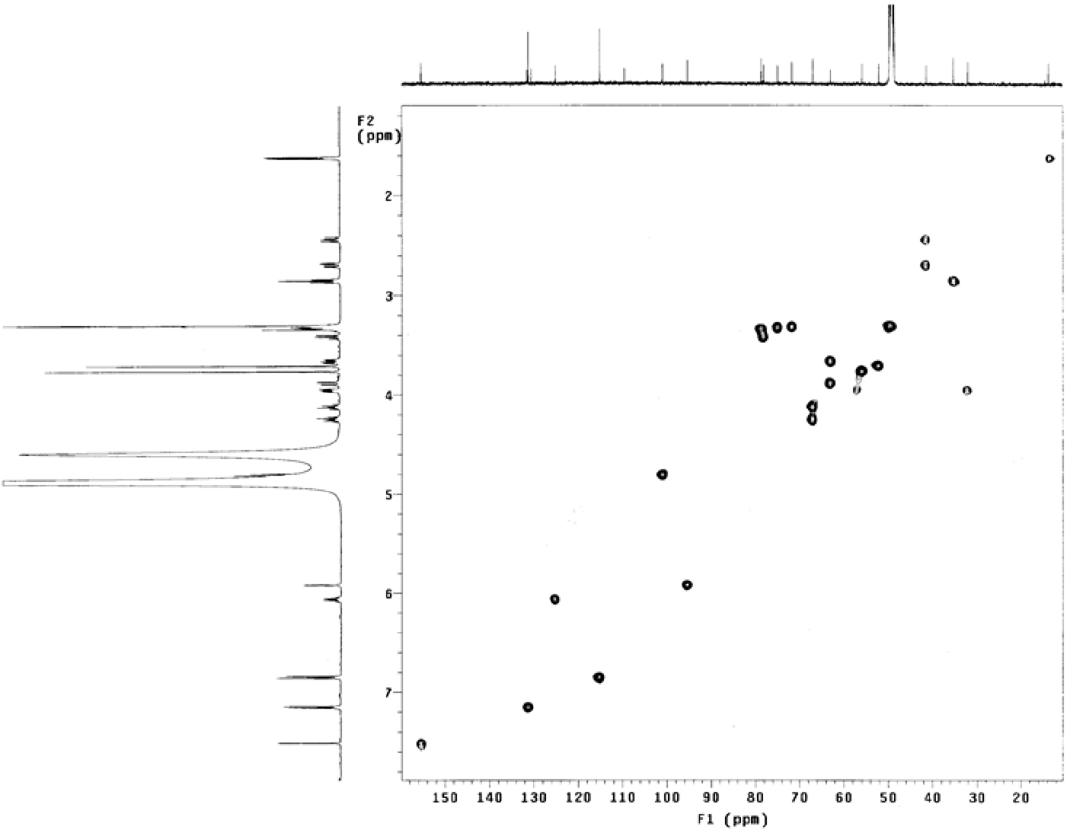

Figure S9. HSQC spectrum of 1.

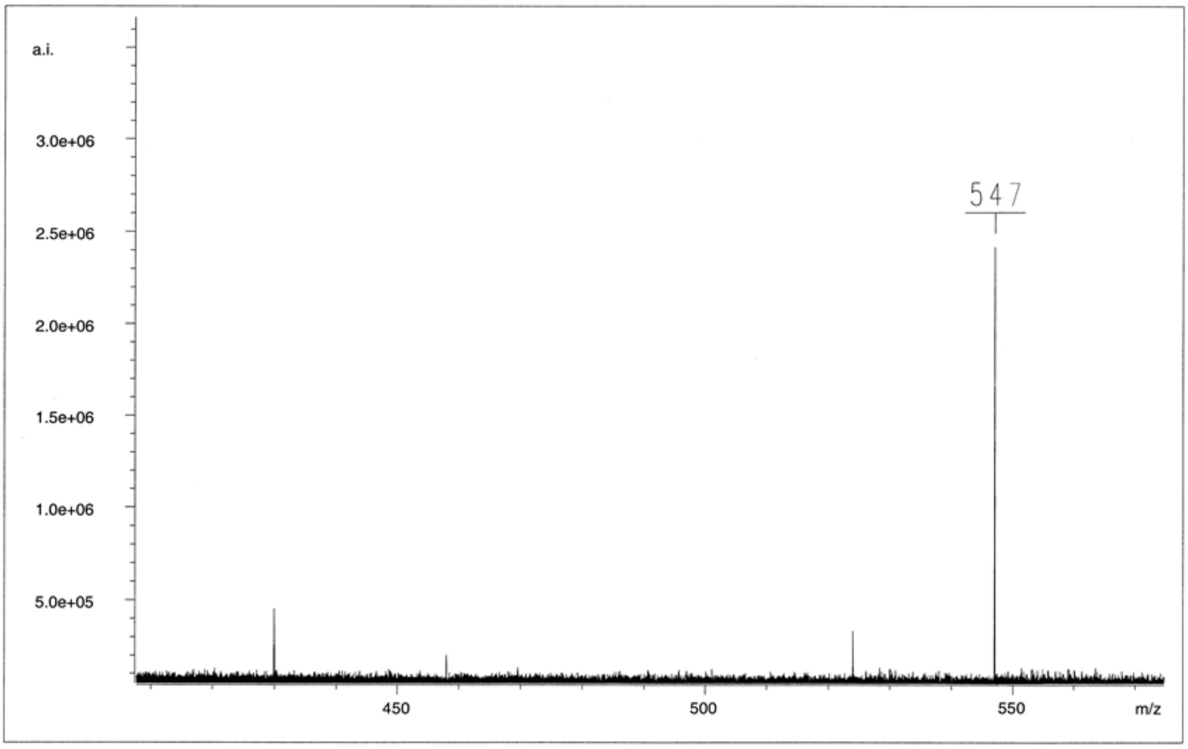

Figure S10. ESI-MS spectrum of 2.

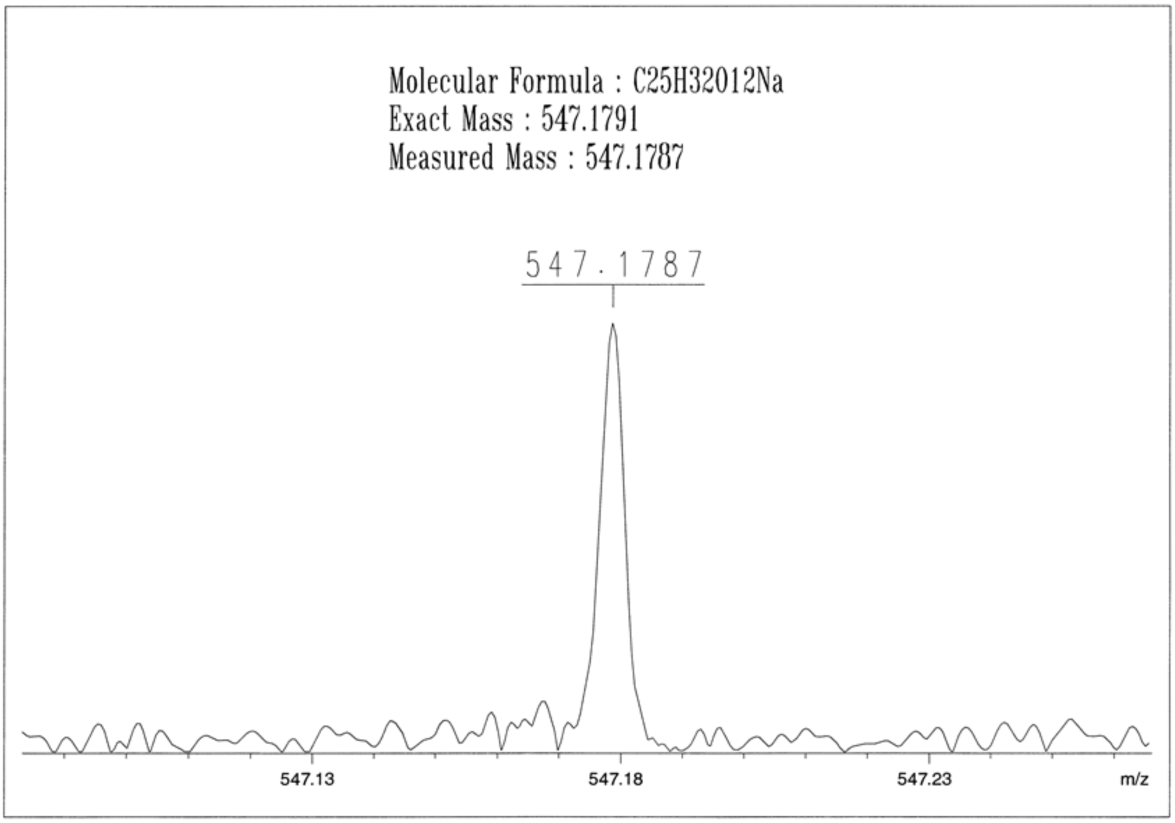

Figure S11. HR-ESI-MS spectrum of 2.

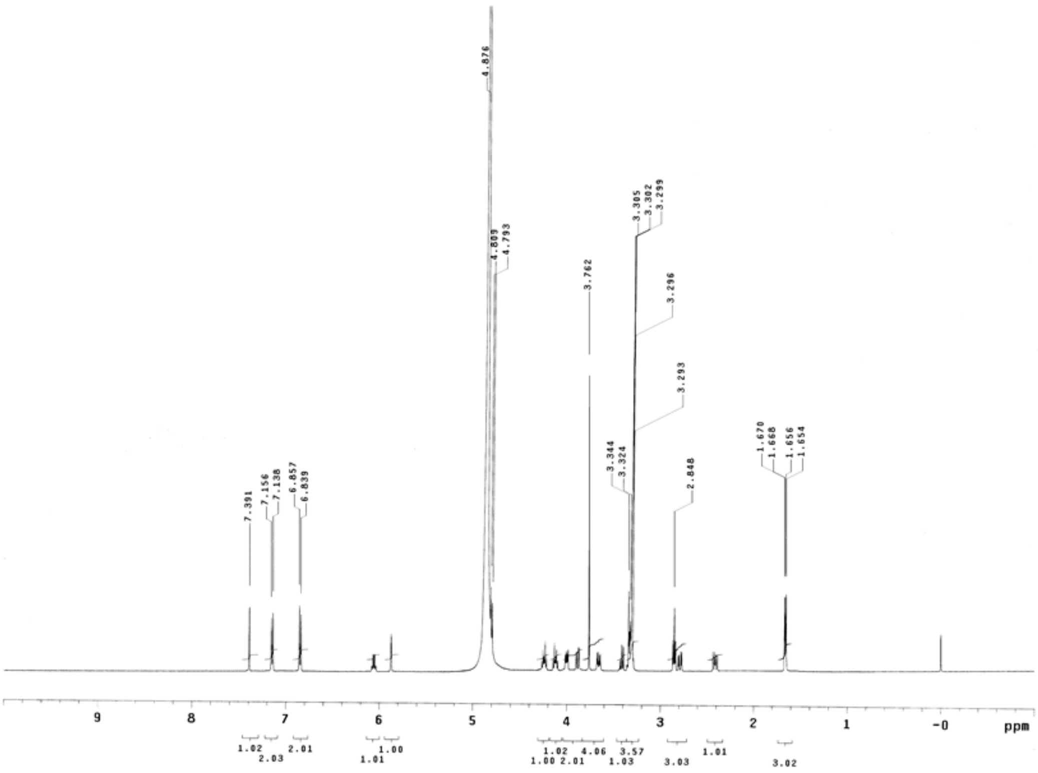

Figure S12. ^{1}H-NMR spectrum (CD_3OD, 500 MHz) of 2.

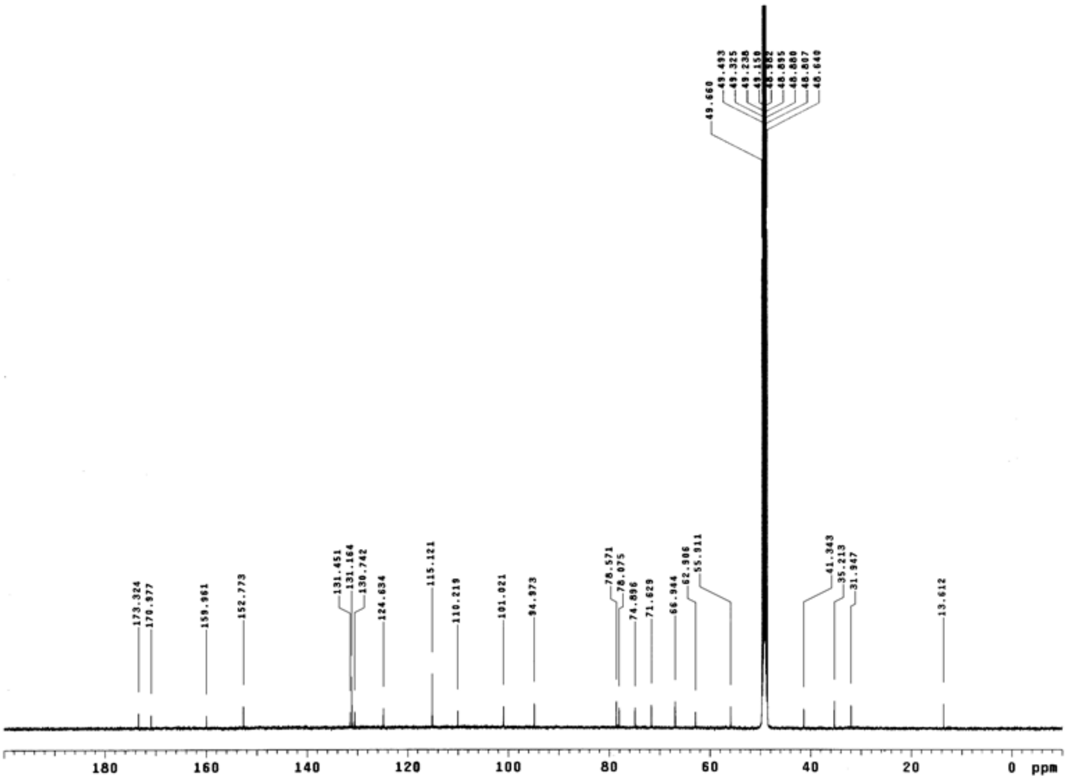

Figure S13. ^{13}C-NMR spectrum (CD_3OD, 125 MHz) of 2.

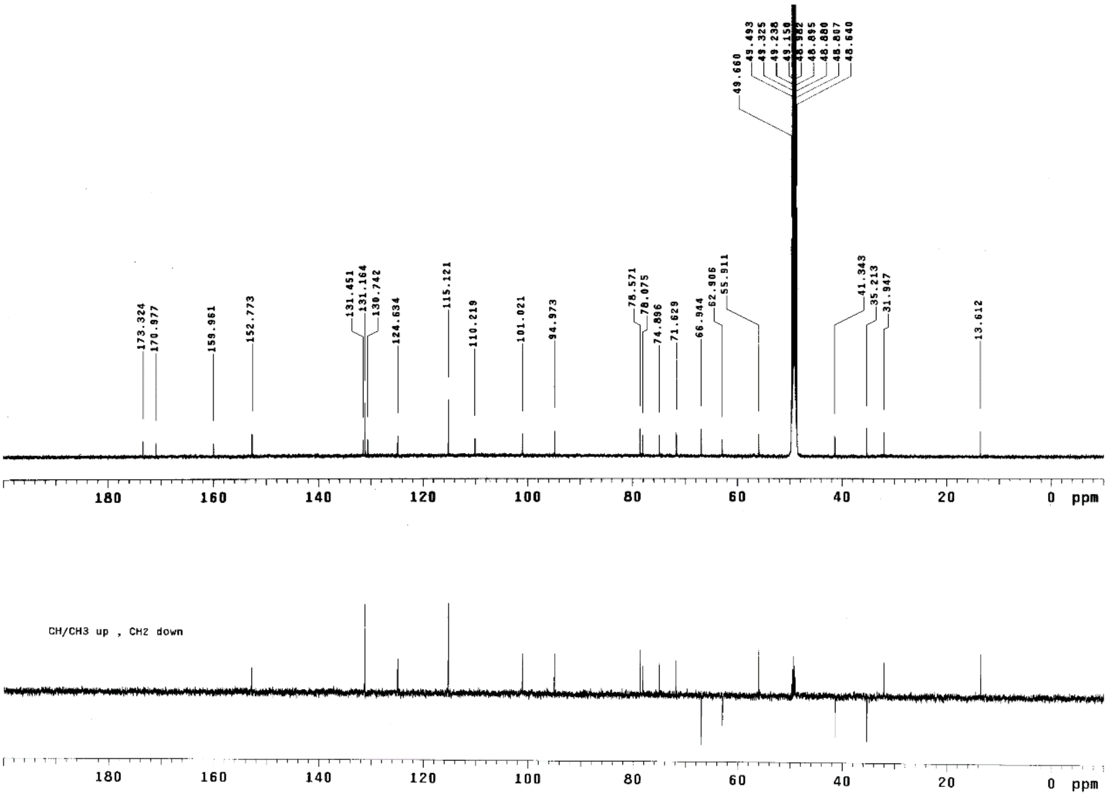

Figure S14. DEPT spectrum (CD_3OD, 125 MHz) of 2.

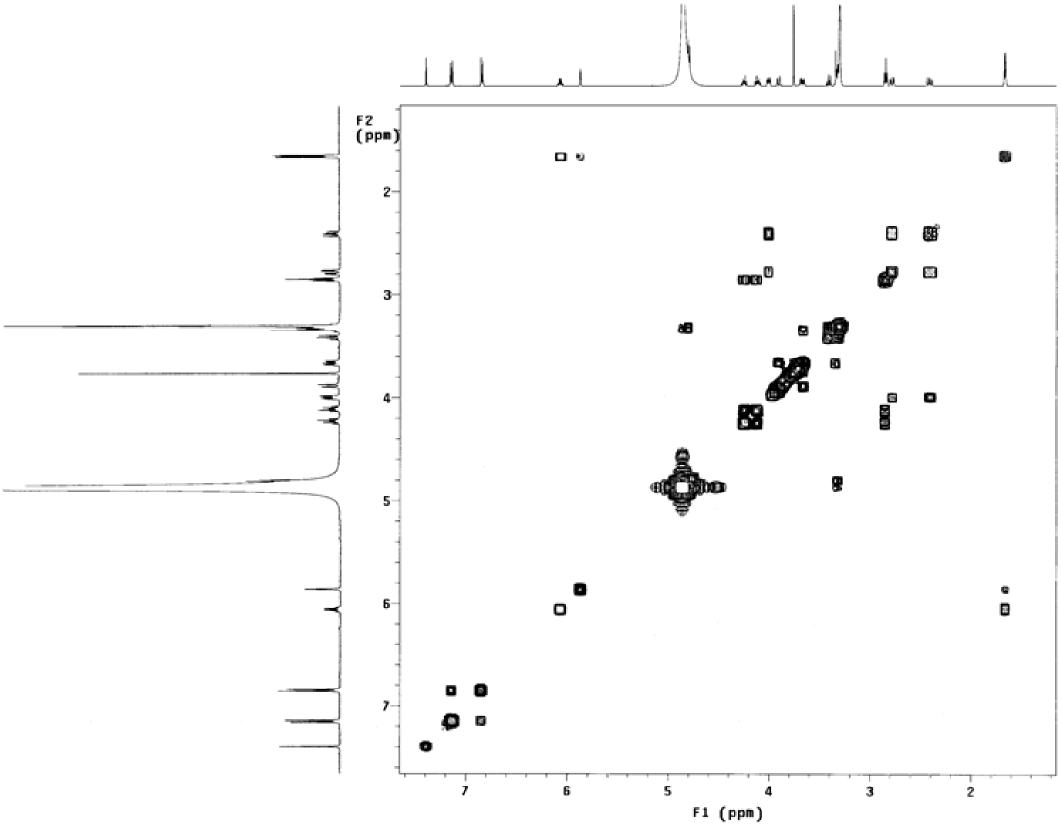

Figure S15. ^{1}H–^{1}H COSY spectrum of 2.

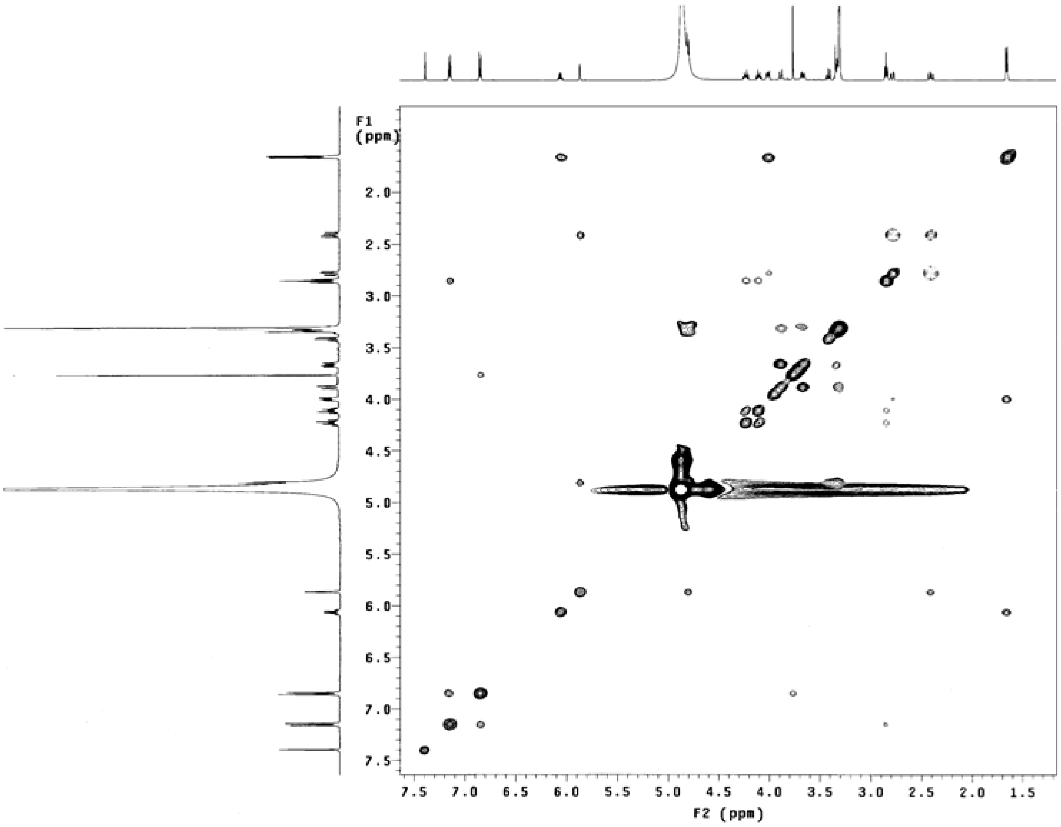

Figure S16. NOESY spectrum of 2.

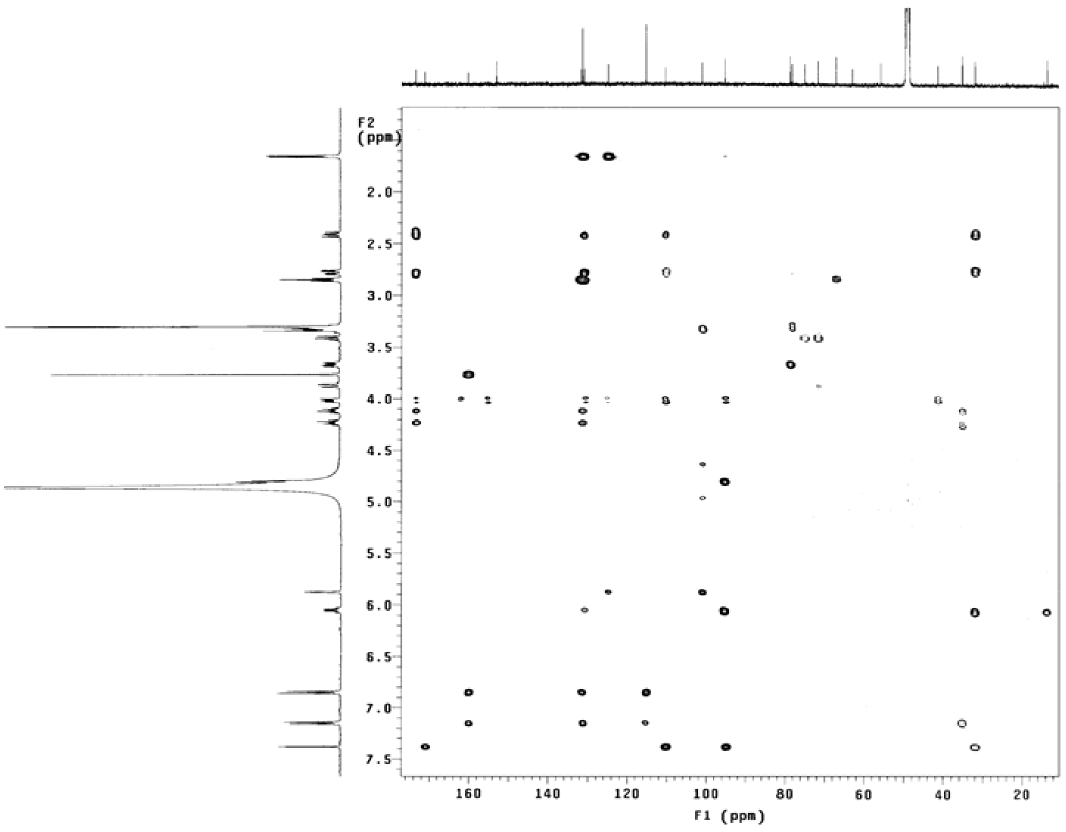

Figure S17. HMBC spectrum of 2.

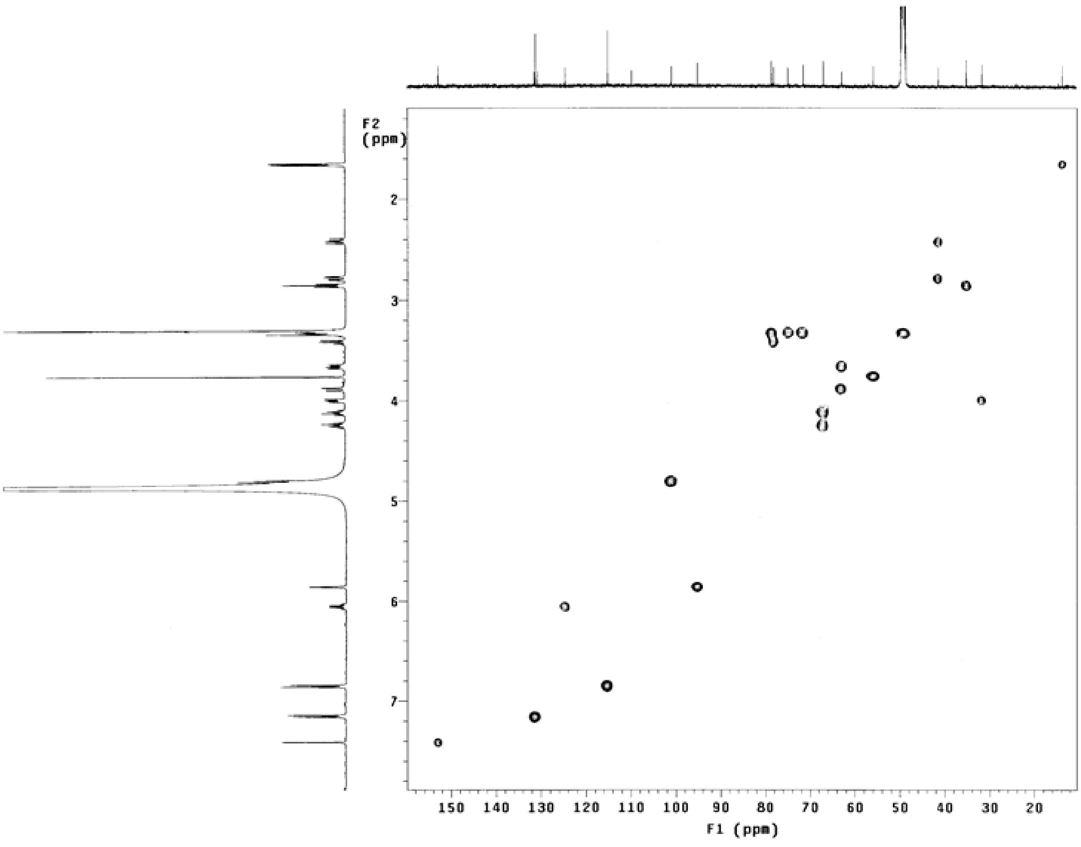

Figure S18. HSQC spectrum of 2.

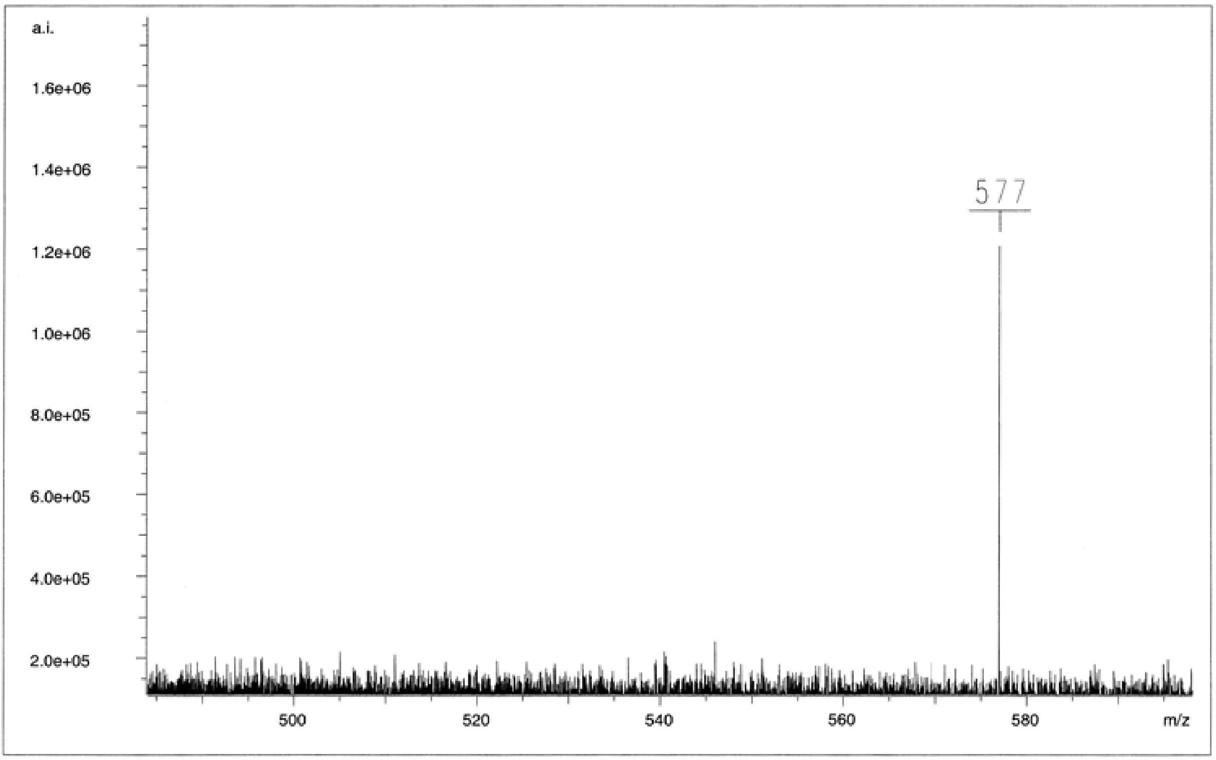

Figure S19. ESI-MS spectrum of 3.

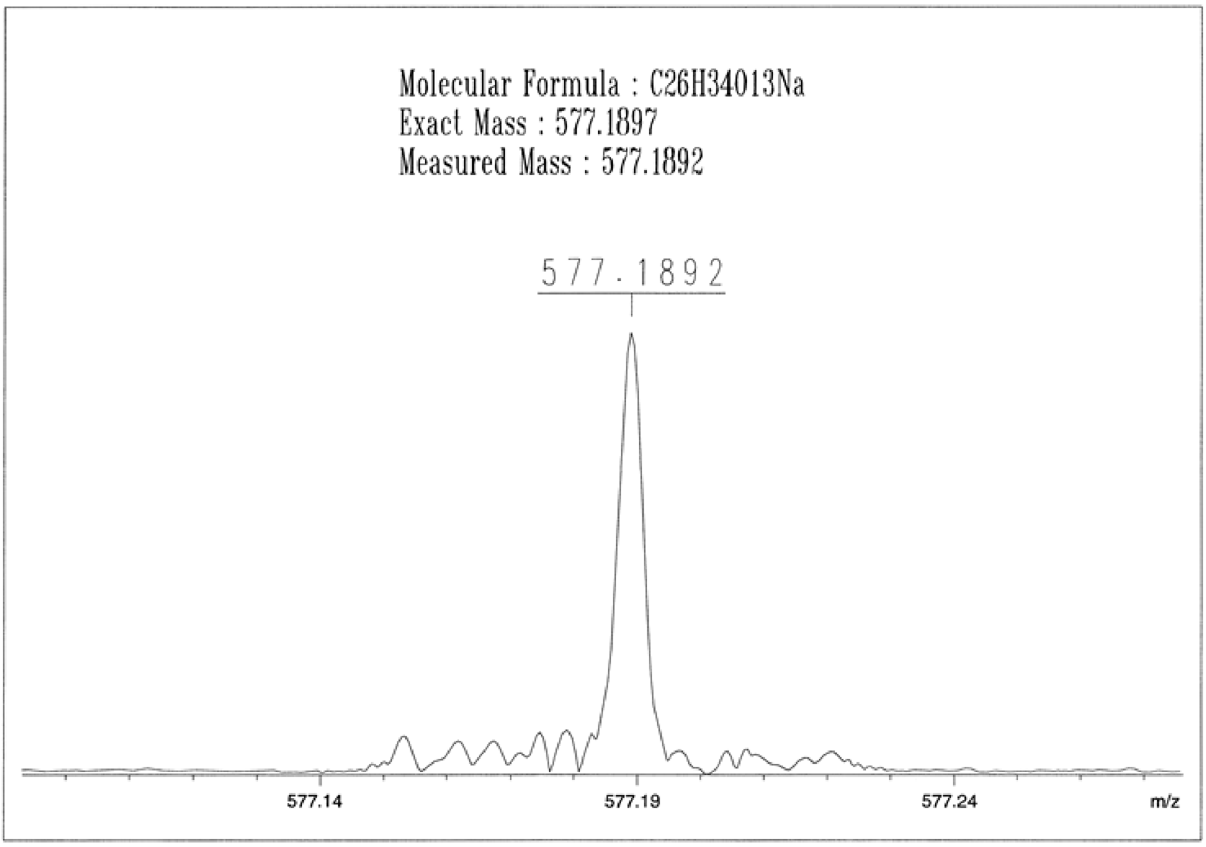

Figure S20. HR-ESI-MS spectrum of 3.

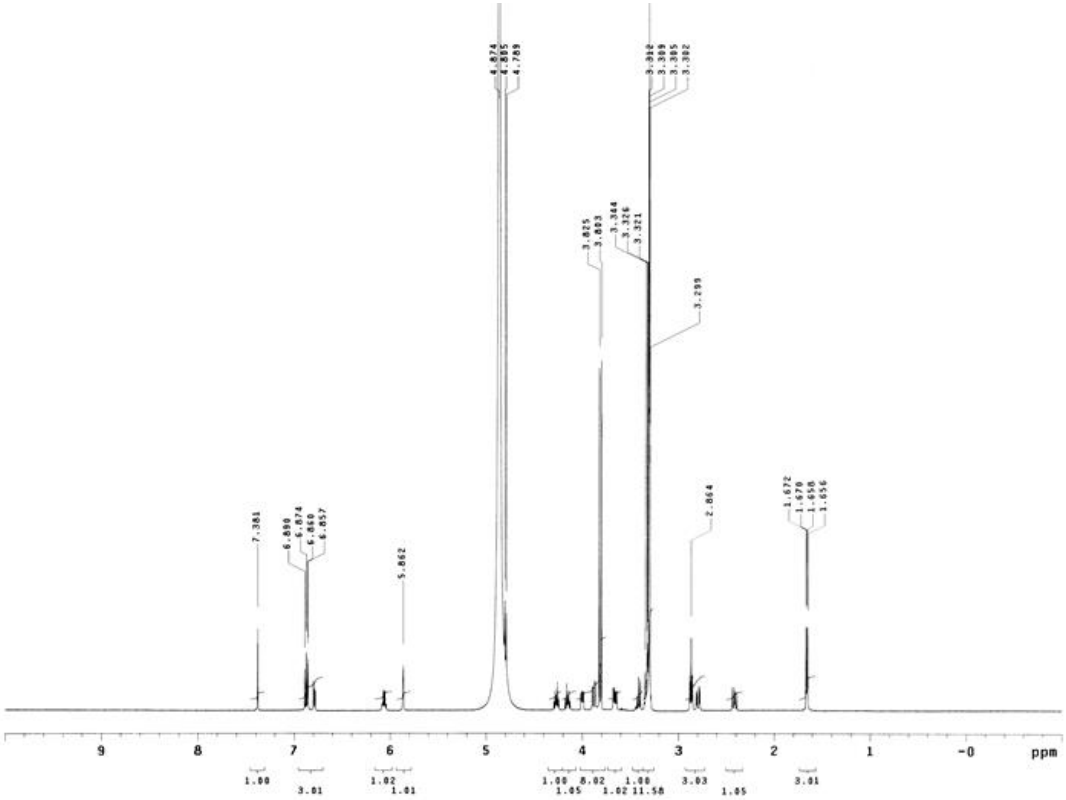

Figure S21. ^{1}H-NMR spectrum (CD_3OD, 500 MHz) of 3.

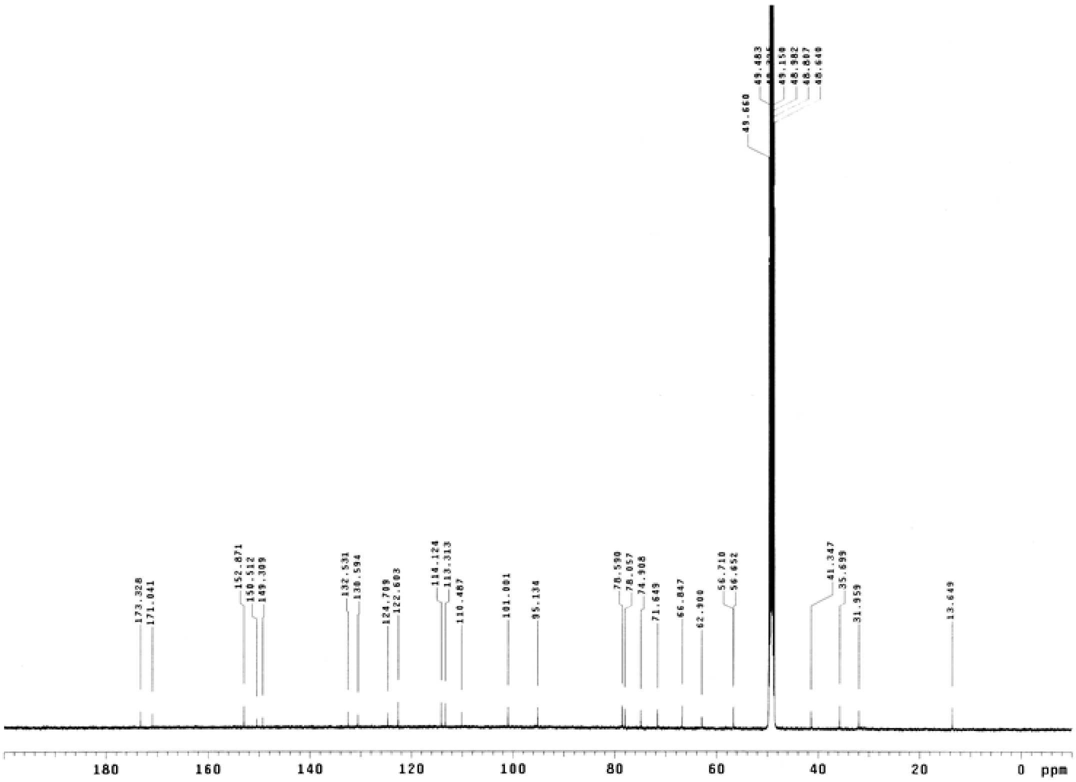

Figure S22. ^{13}C-NMR spectrum of 3 (CD_3OD, 125 MHz) of 3.

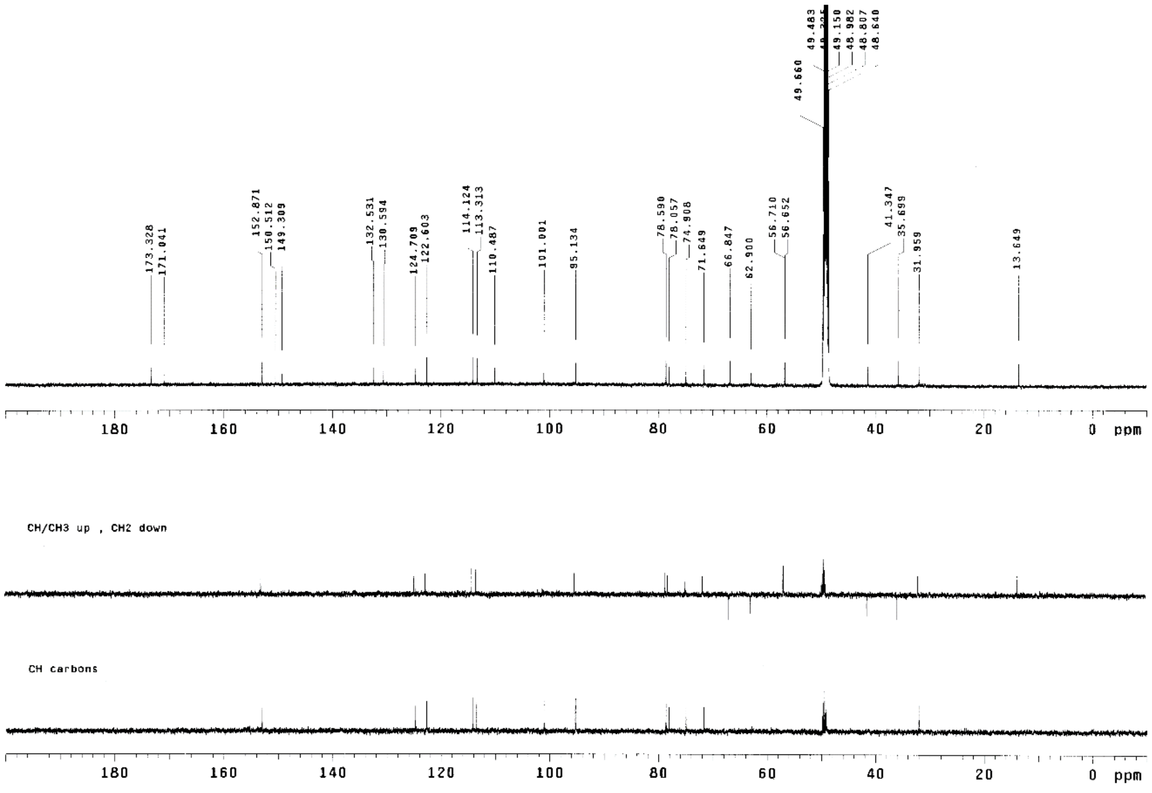

Figure S23. DEPT spectrum of 3 (CD_3OD, 125 MHz) of 3.

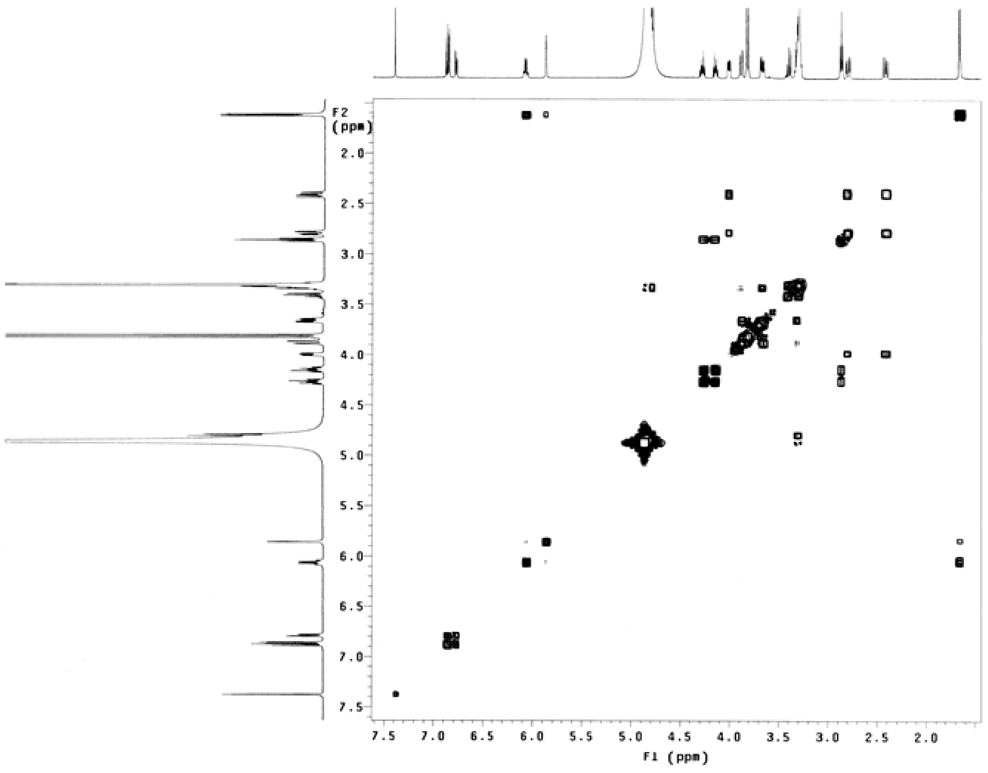

Figure S24. ^1H–^1H COSY spectrum of 3.

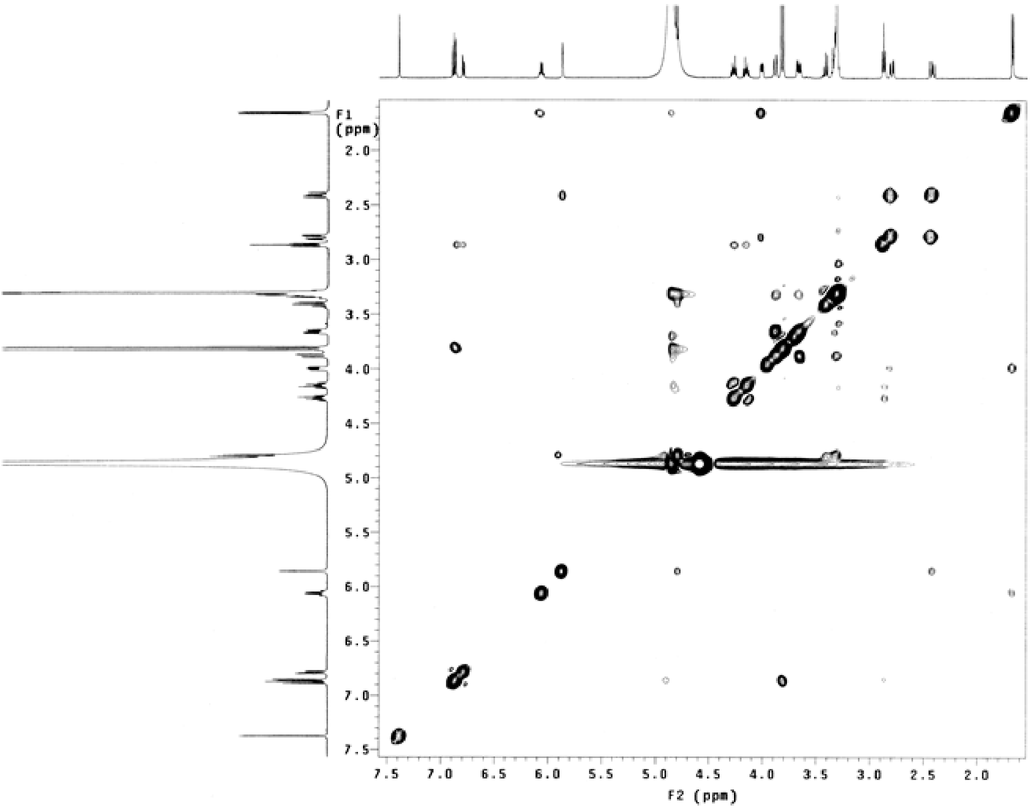

Figure S25. NOESY spectrum of 3.

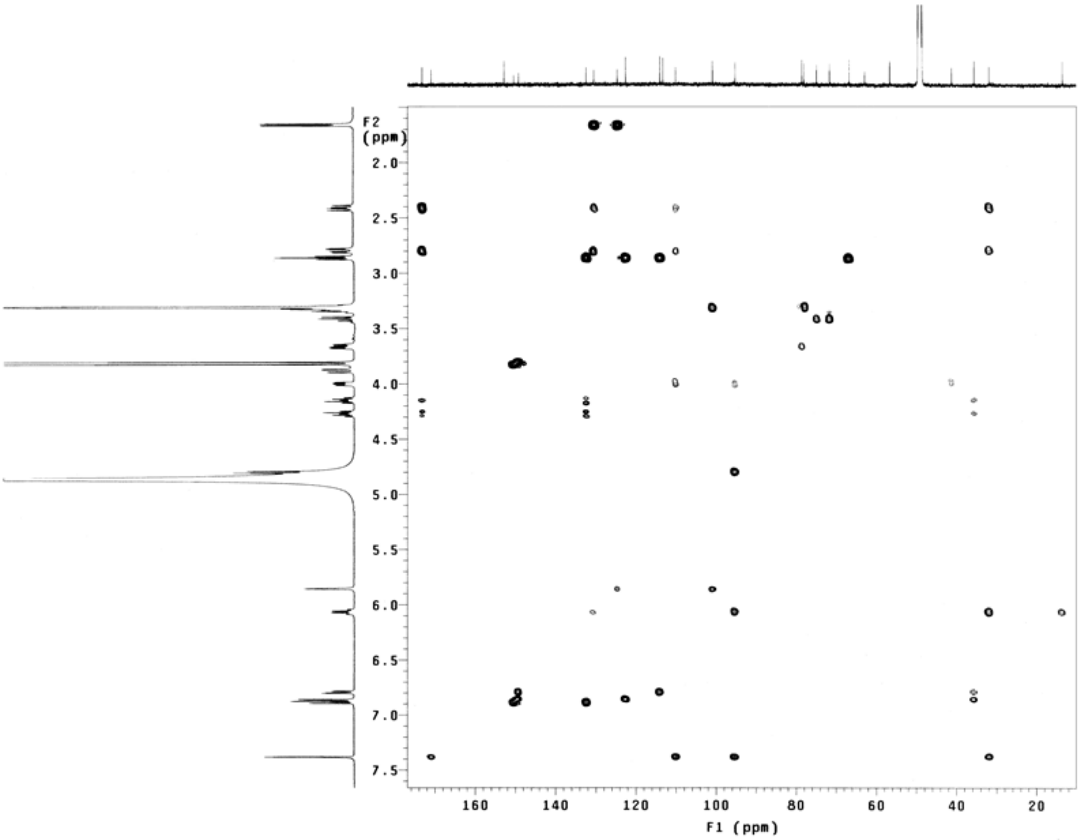

Figure S26. HMBC spectrum of 3.

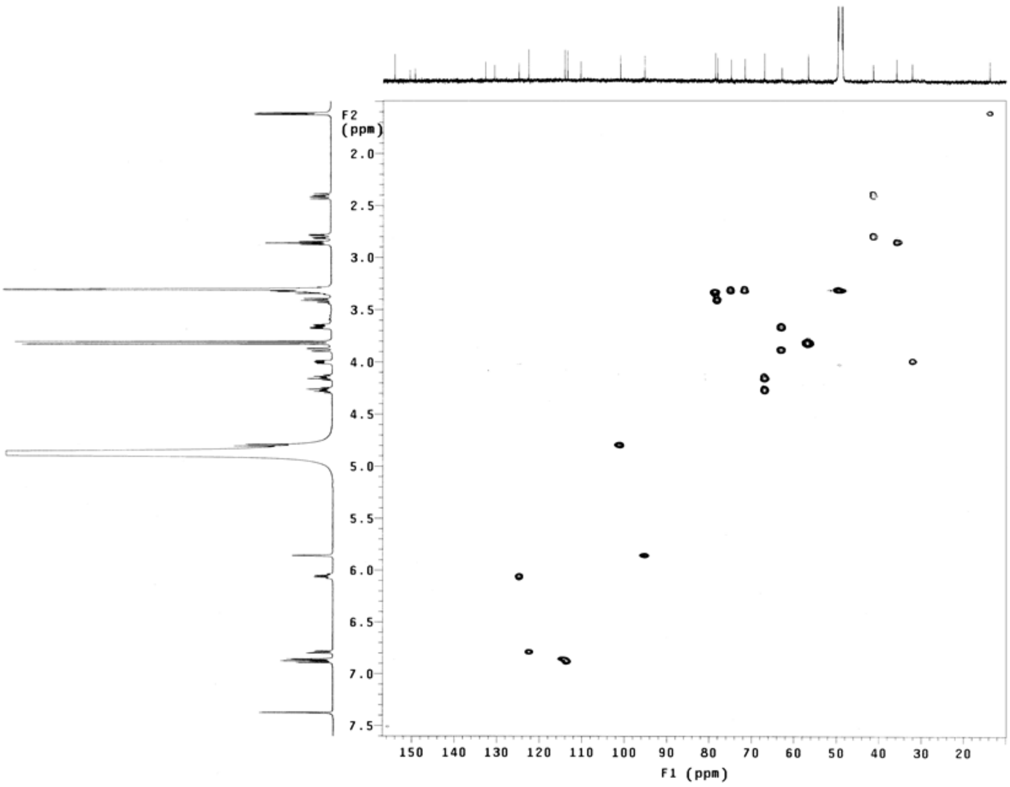

Figure S27. HSQC spectrum of 3.
